# The Effects of a High-Fat/Cholesterol Diet on the Intestine of Rats Were Attenuated by *Sparassis latifolia* Polysaccharides

**DOI:** 10.17113/ftb.60.04.22.7561

**Published:** 2022-12

**Authors:** Xin Wei, Yuan Gao, Feier Cheng, Shaojun Yun, Mingchang Chang, Jinling Cao, Yanfen Cheng, Cuiping Feng

**Affiliations:** 1 College of Food Science and Engineering, Shanxi Agricultural University, Taigu 030801, Shanxi, PR. China; 2 Shanxi Research Station for Engineering Technology of Edible Fungi, Taigu 030801, Shanxi, PR China

**Keywords:** *Sparassis latifolia* polysaccharides, intestinal cholesterol metabolism disorder, gut microbiota, short-chain fatty acids

## Abstract

**Research background:**

*Sparassis latifolia* polysaccharides can regulate lipids and cholesterol in serum and liver. However, little is known about the regulation mechanism of the polysaccharides on cholesterol metabolism and especially the causal relationship with gut microbiota regulation. This study will provide a theoretical basis for the cholesterol-lowering mechanism of *S. latifolia* polysaccharides and further development of functional foods.

**Experimental approach:**

In this study, we investigated how the regulation mechanism of *Sparassis latifolia* polysaccharides affects intestinal cholesterol metabolism in high-fat and high-cholesterol diet-fed rats. Briefly, enzymatic colorimetric microplate assay was used to determine the concentration of faecal bile acid. Gas chromatography-mass spectrometry was used to detect the content of cholesterol and alcohol in faeces. Haematoxylin and eosin staining method was applied to observe the changes in the structure of the small intestine tissue. The related gene expressions in jejunum and ileum were detected by real-time fluorescent quantitative polymerase chain reaction. The related protein expressions in jejunum were studied by using Western blot. High-throughput sequencing was used to detect the intestinal flora changes of the caecal contents. Gas chromatography-mass spectrometry was applied to detect the concentration of short-chain fatty acids in the caecal content.

**Results and conclusions:**

The results showed that *Sparassis latifolia* polysaccharides could improve the intestinal morphological structure and physiological indices in rats fed high-fat and high-cholesterol diet. Moreover, it could improve intestinal cholesterol metabolism disorder induced by high-fat and high-cholesterol diets *via* the reduction of the expression of HMGCR, NPC1L1, ACAT2, MTP, ASBT and IBABP mRNA or protein, increasing *ABCG8* mRNA expression. In addition, it could also increase the relative abundance of *Bacteroides*, *Butyricicoccus*, *Parabacteroides*, *Parasutteerella* and *Alloprevotella* and the short-chain fatty acid concentration, to comprehensively regulate the intestinal cholesterol metabolism. The metabolomics analysis found that *Sparassis latifolia* polysaccharides could affect lipid, carbohydrate and other related metabolites. Some biomarkers associated with cholesterol metabolism correlated significantly with the abundance of specific intestinal microbiota.

**Novelty and scientific contribution:**

These findings indicate that *Sparassis latifolia* polysaccharides could attenuate intestinal cholesterol metabolism disorder, correlating with modulating gut microbiota and improving host metabolism. They provide theoretical support for the development of *Sparassis latifolia* as a new food resource.

## INTRODUCTION

In recent years, the number of overweight and obese people has increased significantly with the increasing proportion of high-fat and high-cholesterol foods in people’s daily diets (*1*). Cholesterol plays an important role in maintaining the normal metabolism of the body. It can be converted into steroid hormones, bile acids and vitamin D; however, excessive intake of cholesterol can lead to its accumulation in blood and metabolic imbalance in the body, causing chronic diseases such as hypercholesterolaemia and atherosclerosis (*2*). Increased total cholesterol and low-density lipoprotein cholesterol in serum are both considered to be the vital risk factors for coronary heart disease, atherosclerosis and stroke (*3*). Cholesterol-related complications are the main causes of morbidity and mortality around the world. Successful regulation of cholesterol metabolism has been believed to prevent the accumulation of cholesterol in the body and thereby reduce the risk of these complications.

The intestinal microbiota could lower intestinal absorption of dietary and biliary cholesterol (*3*). In the enterohepatic circulation, cholesterol in the lumen is transported by NPC1L1 across the apical membrane of the intestinal cells, where the cholesterol absorption can be decreased by the specific bacteria including *Lactobacillus* strains and prebiotic fibre through NPC1L1 transcriptional downregulation (*3*). In addition, the uptake of both dietary and biliary cholesterol by the LDL receptor and the endogenous synthetic cholesterol in the liver are drastically downregulated by the intestinal microbiota. The cholesterol-lowering probiotics in the intestines can reduce the cholesterol content in the small intestine, the absorption of cholesterol by the epithelial cells of the small intestine and the cholesterol concentration in the blood by degrading the bound cholate into the unbound cholate and absorbing cholesterol directly (*4*). However, the dysregulation of cholesterol enterohepatic cycle caused by microbiota depletion leads to an increase in the cholesterol concentration in each compartment, notably in the plasma (*3*). On the other hand, the metabolites of the intestinal flora can regulate the cholesterol metabolism of the host. For example, the short-chain fatty acids (SCFAs) including acetic, propionic and butyric acids, *etc.*, produced by carbohydrate degradation by microorganisms, are one of the most important markers of intestinal flora metabolites that can provide energy for metabolism, regulate intestinal pH and inhibit the reproduction of harmful bacteria. Acetic acid can lower cholesterol, dilate the blood vessels and delay atherosclerosis. Propionic acid is an energy source for liver metabolism and can inhibit the synthesis of cholesterol (*5*). It can inhibit the synthesis of cholesterol in liver tissues and the expression of HMG-CoA reductase mRNA. It has been reported that acetate, propionate, and butyrate could reduce plasma total cholesterol in hypercholesterolaemic hamsters by increasing the excretion of bile acids in faeces and promoting the cholesterol intake in the liver from the blood (*6*).

The interest in using the polysaccharides to regulate cholesterol metabolism is growing. A large number of studies have shown that intake of dietary polysaccharides can shape the intestinal flora, promote the growth and proliferation of intestinal probiotics, and regulate and maintain their normal physiological activities including lowering lipid and cholesterol content (*3*). Zhao *et al.* (*7*) conducted a research on the fungi of the genus *Sparassis* in East Asia and found that, according to the morphological evidence and polygenic DNA sequence analysis, there are three species in East Asia, namely, the new species of *S. subalpina*, *S. cystidiosa* f. *flabelliformis* and *S. latifolia*. The fungi of the genus *Sparassis* are mainly distributed in the northern temperate zone. Their fruiting bodies are tender, delicious and have a great effect on the prevention of diseases such as hyperglycaemia. *Sparassis latifolia* is rich in high-molecular-mass polysaccharides, among which the content of β-glucan can reach 39.3-43.6% of dry mass. A previous study found that *S. latifolia* could maintain the expressions of TLR4 and MyD88 in the small intestine to enhance the production of tumour necrosis factor (TNF) induced by exhaustive exercise (*8*). The *S. latifolia* polysaccharides fermented by lactic acid bacteria could enhance the gene expressions of immune-related factors. They have the ability to activate the body's white blood cells and related immune system. Their anti-inflammatory activity was correlated with the TLR-mediated NF-κB and MAPK signalling pathways in RAW 264.7 macrophages stimulated by lipopolysaccharides (*9*). In addition, *S. latifolia* polysaccharides can reduce lipid metabolism-related biochemical indicators of serum and liver, and regulate body amino acid metabolism (*10*). The previous study of our group proved that *Sparassis* polysaccharides can improve dyslipidaemia in hyperlipidaemia rats, significantly reduce serum total cholesterol concentration, and regulate lipid metabolic processes such as glutamate and glutamine (*11*). Although *S. latifolia* polysaccharides could regulate lipids and cholesterol in serum and liver, little is known about their regulation mechanism of cholesterol metabolism and especially the causal relationship with gut microbiota regulation. Therefore, in this study, a high-fat and high-cholesterol diet rat model was established to investigate the effects and mechanisms of *S. latifolia* polysaccharides on rat cholesterol metabolism by studying the excretion of cholesterol and related metabolites in faeces, the expressions of genes and proteins related to cholesterol metabolism in the small intestine, and the changes of gut microbiota and their metabolites SCFAs in the caecum, which will provide a theoretical basis for the cholesterol-lowering mechanism of *S. latifolia* polysaccharides and further development of functional foods.

## MATERIALS AND METHODS

### Preparation of Sparassis latifolia polysaccharides

The fruiting bodies of *Sparassis latifolia* were provided by Taihe edible mushroom cultivation base Co., Ltd., Qingxu, PR China. *S. latifolia* polysaccharides were prepared according to our previous study (*12*). Briefly, the pulverized *S. latifolia* fruiting bodies were added to distilled water at a ratio of 1:40 (*m/V*) and cultured in a water bath at 75 °C for 3 h after the ultrasound (supersonic machine SB25-12DT; Kunshan Ultrasonic Instruments Co., Ltd., Shanghai, PR China) treatment at 1200 W for 20 min, during which the mixture was stirred regularly. After centrifugation (high-speed refrigerated centrifuge 5417R; Eppendorf, Hamburg, Germany) at 280×*g* for 15 min, the supernatant was concentrated to become viscous (rotary evaporator RV10; IKA® Werke GmbH & Co, Staufen, Germany), 3 times volume of absolute ethanol was added, and shaken thoroughly at 4 °C overnight. Then, the precipitate was collected and washed with acetone and ether in a fume hood until the washing solution was clear. Afterwards, the precipitate was placed in a beaker and heated in an electric furnace (DK-98-ll; Tianjin Taisite Instrument Co., Ltd., Tianjin, PR China) until melted. After cooling, an appropriate amount of neutral protease was added to the precipitate and stirred regularly in a water bath at 45 °C for 8 h. After inactivation of the enzyme and centrifugation, 1/3 volume of the Sevag reagent (*V*(chloroform):*V*(*n*-butanol)=5:1; Merck, Darmstadt, Germany) was added to the supernatant, stirred for 30 min (magnetic stirrer LKTC-B1-T; Xingdong Xinrui Instrument Factory, Jintan, PR China) and centrifuged again. The process was repeated one more time. The supernatant was purified with macroporous resin (HZ-830; Beijing Solaibao Technology Co., Ltd, Beijing, PR China) and freeze-dried *in vacuo* (vacuum freeze dryer Heto PowerDry LL3000; Thermo Fisher Scientific, Waltham, MA, USA) to obtain the *S. latifolia* polysaccharides. Through structural identification, the relative molecular mass of the polysaccharides was found to be from 215 Da to 393 kDa, their main chain was β-(1→3)-d-glucan, and the branch was β-(1→6)-d-glucan, which were composed of *n*(galactose):*n*(glucose):*n*(xylose):*n*(mannose):*n*(fructose)=10.55:24.76:2.51:5.64:1.3.

### Experimental design

Fifty healthy female specific pathogen-free (SPF) Sprague Dawley rats (6 weeks old, weighing 180-220 g) were purchased from Beijing Weitong Lihua Laboratory Animal Technology Co., Ltd, Beijing, PR China, and placed in an animal house at 24 °C, 56% humidity and a 12 h light-dark cycle for one week to adapt to the environment. After adaptation, the rats were randomly divided into five groups: the negative control group (NC group), the high fat/cholesterol model group (HFC group), the group receiving low dose of polysaccharides (LD group), the group receiving medium dose of polysaccharides (MD group) and the group receiving high dose of polysaccharides (HD group). Rats in the NC group were given a basic diet (Beijing Weitong Lihua Laboratory Animal Technology Co., Ltd, Beijing, PR China) (Table S1) and the rats in the HFC, LD, MD and HD groups were given high-fat and high-cholesterol diet prepared in our laboratory (88.8% basal diet, 10% lard, 1% cholesterol and 0.2% pig bile salt). The rats in LD, MD and HD groups were administered intragastrically 100, 200, 400 mg/(kg·day) of *S. latifolia* polysaccharides, and the rats in NC and HFC groups were given the same volume of normal saline by gavage for 8 weeks, respectively. According to our previous study, *S. latifolia* polysaccharides were safe for the animal intestines (*13*). Rats had *ad libitum* access to food and water during the adaptation and experiment period under SPF conditions. After fasting for 12 h, they were anesthetized with ether, and then sacrificed by cervical dissection. All work on animals was authorized by Institutional Animal Care and Use Committee of Shanxi Agricultural University, PR China.

### Determination of total bile acid, cholesterol and coprostanol in rat faeces

For the last three days of feeding, the rats were placed in a clean cage, the faeces were collected, vacuum freeze-dried, pulverized, and stored under dry conditions for testing. The faecal sample (0.1 g) in each group was mixed with 9 times volume of normal saline and centrifuged (5417R; Eppendorf) at 1500×*g* to obtain the supernatant. The total bile acid content in the faeces was measured using total bile acid kit (Nanjing Jiancheng Bioengineering Institute, Nanjing, PR China). The neutral sterols in rat faeces samples were extracted and derivatized, and the contents of two neutral sterols (cholesterol and coprostanol) were detected by gas chromatography-mass spectrometry (GC-MS). Briefly, 50 μL of 1 mg/mL 5α-cholestane in chloroform solution (Shanghai Aladdin Biotechnology Co., Ltd., Shanghai, PR China) as the internal standard and 2 mL of *V*(methano):*V*(chloroform)=1:2 were added to 0.1 g faecal sample, shaken well and layered. After removing the lower layer of liquid, the mixture was dried with nitrogen (nitrogen evaporator N-EVAP 111; Organomation, Berlin, MA, USA), 2 mL of 1 mol/L methanol KOH solution (Sinopharm Chemical Reagent Beijing Co., Ltd, Beijing, PR China) were added, and cultured in a water bath at 50 °C for 60 min. Then, 2 mL of ultrapure water and 5 mL of *n*-hexane (Merck) were added, and the mixture was dried with nitrogen. Afterwards, the precipitate was dissolved in 100 μL of pyridine solution and filtered into a sample bottle through a 0.22-μm filter, 50 μL of silanization reagent (Shanghai Aladdin Biotechnology Company) were added, and the bottle was sealed to react for 30 min at room temperature. Finally, the solution was put in an ice water bath to stop the reaction, and the mass fractions of cholesterol and coprostanol in faeces were detected by GC-MS analysis (Trace^TM^ 1600; Thermo Fisher Scientific).

### Histopathological observation of rat ileum

The rats were dissected, and the ileum tissue about 1 cm away from the caecum was collected, cleaned and placed in neutral formaldehyde fixative. After being fixed, the ileum tissues were embedded in paraffin, cut into 5 μm sections by microtome (Leica RM2265; Leica Microsystems Trading Co., Ltd., Wetzlar, Germany). Deparaffinized ileum tissue sections were stained with hematoxylin and eosin (Beijing Solabo Technology Co., Ltd, Beijing, PR China). Subsequently, the sections were observed by an optical microscope (model IX3; Beijing Shunneng Electronic Instrument Co., Ltd., Beijing, PR China) at 200×. The ratio of villus length to crypt depth was also calculated.

### Determination of the surface area of caecum wall

The caecal wall was washed with distilled water, weighed after drying, wrapped in tin foil and stored at 4 °C. According to the method of Lu *et al.* (*14*), the wall of the caecum was cleaned with normal saline, fixed on a white paper and measured with a standard calibration (cm). After taking a photograph, the picture was printed, cut along the contour of the caecum wall, the length was measured and the cut-out was weighed accurately, as well as 1 square centimetre of paper. The surface area of caecum wall was calculated according to the following equation:

*A*=(*m*_1_/*m*_2_)·*l*^2^ /1/

where *A* is the surface area of caecum wall (in cm^2^), *m*_1_ is the mass of the cut-out picture (in g), *m*_2_ is the mass of the 1 square centimeter of paper (in g) and *l* is the length of a cut-out picture (in cm).

### Determination of the water mass fraction and pH of the caecal content

The caecal tissue was weighed and a part of the content was scraped off and stored at -20 °C. The caecal content was put in an aluminium box, weighed and dried in an oven at 105 °C for 4 h. Then it was put in a glass desiccator to cool down for 0.5 h and weighed again. The operation was repeated until the sample reached a constant mass. The water mass fraction was calculated according to the following equation:

*w*=((*m*_1_-*m*_2_)/*m*)·100 /2/

where *w* is the water mass fraction of caecal content, *m* is the mass of the caecal content (in g), *m*_1_ is the mass of the aluminium box and sample before drying (in g) and *m*_2_ is the mass of the aluminium box and sample after drying (in g).

The caecal content (0.2 g) was put in a 10-mL centrifuge tube, 10 times volume of ultrapure water was added and mixed thoroughly with a vortexer (MX-E; Dalong Xingchuang Experimental Instrument Co., Ltd., Beijing, PR China). After resting for 1 h, the pH of the supernatant was determined by pH meter (ST3100; Ohaus Instruments Co., Ltd., Changzhou, PR China).

### RNA extraction and real time fluorescent quantitative polymerase chain reaction analysis of gene expressions in small intestine

Total RNA was isolated from jejunum and ileum using TRIzol® reagent (Baori Medical Biotechnology Co., Ltd., Beijing, PR China). The primer sequences of related genes used for quantitative real time polymerase chain reaction (qRT-PCR) are given in Table S2 (*15*). The real-time qPCR primers were either obtained from Sigma-Aldrich (Shanghai) Trading Co., Ltd (Shanghai, PR China) or synthesized from validated sequences obtained at PrimerBank (*16*). Expression of key genes were detected using PrimeScript^TM^ RT Master Mix kit (Baori Medical Biotechnology Co., Ltd.) and real-time PCR system (fluorescence quantitative PCR instrument Mx3000P; Stratagene, La Jolla, CA, USA).

### Determination of the expression of proteins related to cholesterol metabolism

The total protein was separated using sodium dodecyl sulfate-polyacrylamide gel electrophoresis (SDS-PAGE) preparation kit (Wuhan Saiweier Biotechnology Co., Ltd. Wuhan, PR China) and then transferred to polyvinylidene difluoride (PVDF) membranes (Wuhan Saiweier Biotechnology Co., Ltd.). The specific protein strips were observed using the chemiluminescence imaging system (Universal Hood II, Bio-Rad Laboratories, Hercules, CA, USA) and analysed using ImageJ program (*17*).

### Gut microbiota analysis

The caecal content was scraped into a sterile tube, frozen in liquid nitrogen and stored at -80 °C for later use. Bacterial DNA was extracted by Beijing Novogene Bioinformatics Technology Co., Ltd. The V4 region of 16S rRNA genes was amplified using specific primer (16S V4: 515F-806R). Sequence analysis was done using UPARSE software (v. 7.0.1001, http://drive5.com/uparse/) (*18*). Sequences with ≥97% similarity were assigned to the same operational taxonomic unit (OTU). Representative sequence for each OTU was screened for further annotation. For each representative sequence, the Silva database (https://www.arb-silva.de/) (*19*) was used based on Mothur algorithm to annotate taxonomic information. In order to study phylogenetic relationship of different OTUs, and the difference of the dominant species in different samples (groups), multiple sequence alignment was conducted using the MUSCLE software (v. 3.8.31, http://www.drive5.com/muscle/) (*20*). Alpha diversity is applied in analysing the complexity of species diversity of a sample through 5 indices, including observed species, Chao1, Shannon, Simpson, ACE. These indices were calculated with QIIME software (v. 1.7.0) (*21*). The analysis of relative abundance of species, cluster and Spearman’s correlations were calculated using QIIME software (v. 1.7.0) (*21*) and visualized by R software, v. 2.15.3 (*22*). For functional analysis, functional abundances from 16S rRNA sequencing data were analysed for the functional prediction of microbiota with Tax4Fun (v. 1.0) (*23*) based on the database of Kyoto Encyclopedia of Genes and Genomes (KEGG) (*24*) and Functional Annotation of Prokaryotic Taxa (FAPROTAX), v. 1.0 (*25*). The correlation heatmap between lipid metabolism biomarkers and intestinal microbiota at the genus level was based on the R software (v. 2.15.3) (*22*).

### SCFA quantification in the caecum content

The SCFAs in the caecum content were quantitatively analysed by gas chromatography (Trace^TM^ 1600; Thermo Fisher Scientific). Briefly, 1 mL of ultrapure water was added to the caecum content (0.2 g) in each group and centrifuged at 59940×*g* (5417R; Eppendorf) for 10 min. Then, the supernatant was mixed thoroughly with 100 μL of 50% sulfuric acid solution and 2 mL of ether (Merck), centrifuged at 59940×*g* for 5 min, and placed in a refrigerator at 4 °C for 30 min. Next, 1 μL of filtered supernatant was injected into a gas chromatograph equipped with a flame-ionization detector and a gas chromatograph column (model DA-FFAP, column length 30 m, inner diameter 0.25 mm, particle size 0.25 μm; Agilent Technology Co., Ltd, Santa Clara, CA, USA). The operation was performed as follows: injection and detector temperature: 230 °C, programmed column temperature: 40 °C for 1 min, 40 to 80 °C at a rate of 10 °C/min, 80 °C for 0.5 min, 10 °C/min to 140 °C, and 140 °C for 0.5 min, followed by 5 °C/min to 180 °C and 180 °C for 5 min. The flow rate of high purity helium was set at 1 mL/min. The content of SCFAs was calculated based on the external standard method using acetic, propionic, isobutyric, butyric, isovaleric and valeric acids as standards (Merck).

### Statistical analysis

Data were expressed as the mean value±standard deviation (S.D.). IBM SPSS statistics (v. 22.0) (*26*) was employed for one-way analysis of variance (ANOVA) with Duncan’s multiple range test. The values at p<0.05 were considered significantly different. All graphs were drawn in GraphPad Prism (v. 8.0) (*27*). In order to study phylogenetic relationship of different OTUs, and the difference of the dominant species in different samples (groups), multiple sequence alignment was conducted using the MUSCLE software (v. 3.8.31, http://www.drive5.com/muscle/) (*20*).

## RESULTS AND DISCUSSION

### Influence of S. latifolia polysaccharides on the mass gain of rats fed high-fat and high-cholesterol diet

**Fig. 1:**
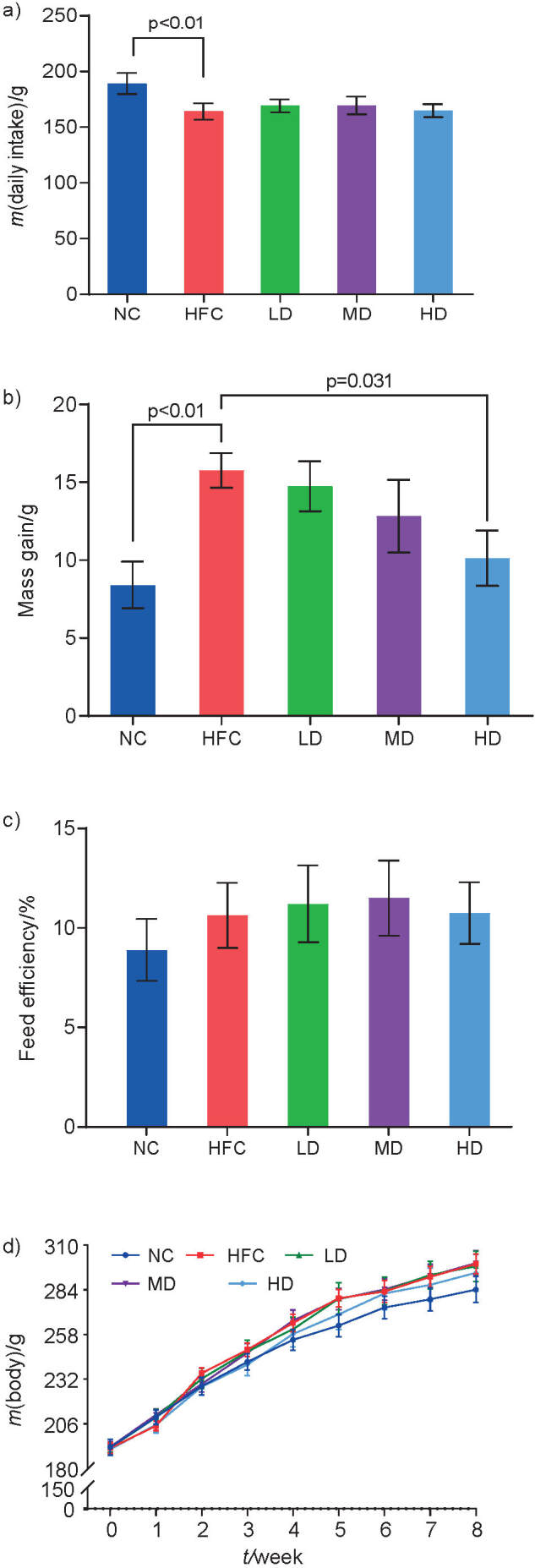
Influence of *Sparassis latifolia* polysaccharide on rat: a) food intake, b) mass gain, c) feed efficiency and d) body mass. NC=normal control group, HFC=high-fat and high-cholesterol diet group, LD=low dose group, MD=medium dose group, HD=high dose group. The significant differences were at p<0.05, highly significant differences were at p<0.0

As shown in Fig. 1, compared with the negative control group (NC group), the food intake in the high fat/cholesterol model group (HFC group) significantly (p<0.05) reduced (Fig. 1a) and the mass gain significantly increased (Fig. 1b). Compared with the HFC group, the mass gain significantly decreased by 35.7% in the group receiving high dose of polysaccharides (HD group). Compared to NC group, the feed efficiency increased in all other groups, but the difference was not significant (Fig. 1c). During the experiment, the body mass of rats in each group showed an increasing trend with the prolonged feeding time (Fig. 1d). Food intake of rats in HFC group was significantly reduced compared to the NC group, which was consistent with previous reports (*1*). This may be because the high-fat and high-cholesterol diet is not easy to digest and absorb in the intestine, and the emptying time is longer.

### Influence of S. latifolia polysaccharides on the physiological indices of faeces of rats fed high-fat and high-cholesterol diet

**Fig. 2:**
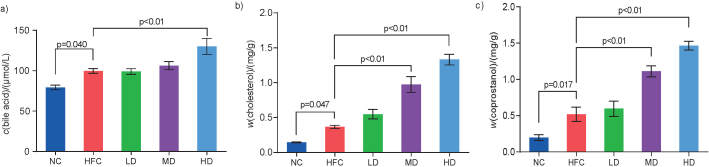
Influence of *Sparassis latifolia* polysaccharides on the contents of: a) total bile acid, b) cholesterol, and c) coprostanol in faeces of rats. NC=normal control group, HFC=high-fat and high-cholesterol diet group, LD=low dose group, MD=medium dose group, HD=high dose group. The significant differences were at p<0.05, highly significant differences were at p<0.01

As seen in Fig. 2, compared with the NC group, the contents of total bile acid, coprostanol and cholesterol in the HFC group significantly increased. Treatment with *S. latifolia* polysaccharides increased the contents of total bile acid, coprostanol and cholesterol of faeces in the groups receiving low and medium dose of polysaccharides (LD and MD groups) and HD groups in a dose-dependent manner compared with the HFC group. The total bile acid content of faeces in the HD group was significantly higher by 30.5% than that in the HFC group (Fig. 2a), and the contents of cholesterol and coprostanol of faeces significantly (p<0.05) increased by 165.8 and 263.7% in the MD, and 114.2 and 182.6% in the HD groups, respectively, compared with the HFC group (Figs. 2b and 2c).

### Influence of S. latifolia polysaccharides on the intestinal physiology of rats fed high-fat and high-cholesterol diet

**Fig. 3:**
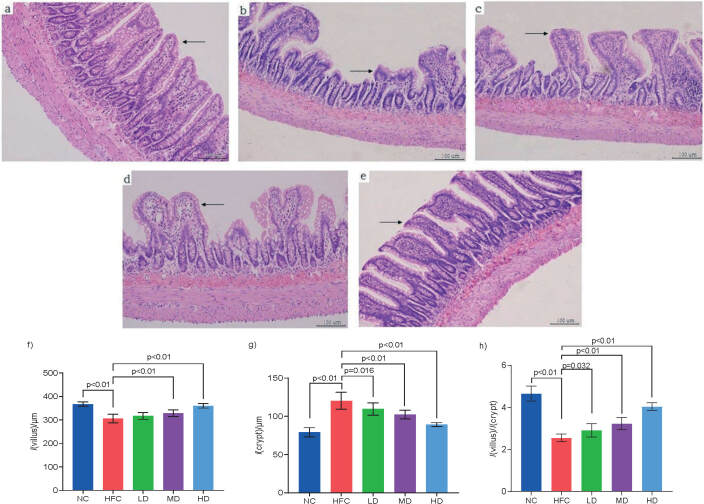
Influence of *Sparassis latifolia* polysaccharides on the structure of ileum: a-e) represent the histopathologic observation of ileum in the NC, HFC, LD, MD and HD group, respectively (→indicated as ileum villi, ←indicated as ileum crypts; 200×), f-g) the villus length and crypt depth of ileum, h) the ratio of villus length to crypt depth. The villus length and crypt depth of jejunum were measured (five samples in each group were taken for five continuous slices in each, and five visual fields were taken in one slice). The villus length was determined from the top of the intestinal villus to the intestinal wall, and the crypt depth was the length of the tubular gland from the root of the villus to the lamina propria. NC=normal control group, HFC=high-fat and high-cholesterol diet group, LD=group receiving a low dose of polysaccharides, MD=group receiving a medium dose of polysaccharides, HD=group receiving a high dose of polysaccharides. The significant differences were at p<0.05, highly significant differences were at p<0.01

Fig. 3a shows the complete epithelium of ileum mucosa in the NC group with clear structure, orderly arranged epithelial cells, the integral gland morphology, and the long and complete villi. Compared with the NC group, the ileum in the HFC group shows obvious pathological changes, serious damage of the intestinal mucosa, that the epithelium had fallen off, the villi were broken and shortened (p<0.05), and crypt depth increased significantly (Fig. 3b). Treatment with *S. latifolia* polysaccharides alleviated the pathological changes of ileum in rats caused by high-fat and high-cholesterol diet. With the increase of *S. latifolia* polysaccharide dose, the structure of mucosa and intestinal epithelial cells tended to be normal, the villi were arranged neatly and similar to the NC group, especially in the intestinal villi of the HD group (Figs. 3c−3e). The villus length significantly (p<0.05) increased in MD and HD groups compared with the HFC group (Fig. 3f). The crypt depth in MD and HD groups significantly (p<0.05) reduced compared with the HFC group (Fig. 3g). However, the ratio of villus length to crypt depth in all groups treated with *S. latifolia* polysaccharides significantly increased (p<0.05) compared with the HFC group (Fig. 3h). In addition, a high-fat and high-cholesterol diet can affect the ability of the small intestine to digest and absorb nutrients by shortening and disarranging the villi of the small intestine, and deepening the crypts, thereby threatening the health of the body. The *S. latifolia* polysaccharides could reduce the damage of high-fat and high-cholesterol diet to the small intestine tissue and protect the integrity of the intestinal structure, increasing the contents of bile acid, cholesterol and coprostanol in the faeces of rats fed high-fat and high-cholesterol diet.

**Fig. 4:**
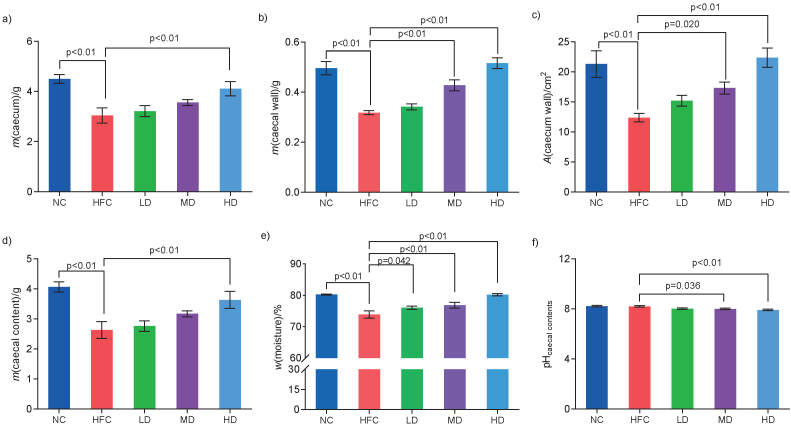
Influence of *Sparassis latifolia* polysaccharides on the caecum and caecal content in rat: a) caecum mass, b) caecal wall mass, c) the surface area of the caecum wall, d) caecal content mass, e) moisture of the caecal content, and f) the pH of the caecal content. NC=normal control group, HFC=high-fat and high-cholesterol diet group, LD=group receiving a low dose of polysaccharides, MD=group receiving a medium dose of polysaccharides, HD=group receiving a high dose of polysaccharides. The significant differences were at p<0.05, highly significant differences were at p<0.01

As shown in Fig. 4, HFC diet reduced significantly (p<0.05) the caecum mass, caecal wall mass, caecal wall surface area, the mass and moisture fraction of the caecal content compared with the NC group. The same parameters increased in a dose-dependent manner in three groups treated with *S. latifolia* polysaccharides and significantly (p<0.05) increased by 35.2, 62.1, 80.6, 31.7 and 8.5%, respectively, in the HD group (Figs. 4a−4e), compared with the HFC group. However, the pH of the caecum content had a downward trend and decreased significantly (p<0.05) in the MD and HD groups, compared with the HFC group.

The results show that *S. latifolia* polysaccharides could significantly improve the morphology of the small intestine, increase the mass and surface area of the caecal wall, the mass and moisture fraction of the caecal content, and reduce its pH, thereby alleviating the damage of the high-fat and high-cholesterol diet to the structure of the intestine in rat, directly or indirectly improving the intestinal environment.

### Influence of S. latifolia polysaccharides on the intestinal cholesterol metabolism of rats fed high-fat and high-cholesterol diet

**Fig. 5:**
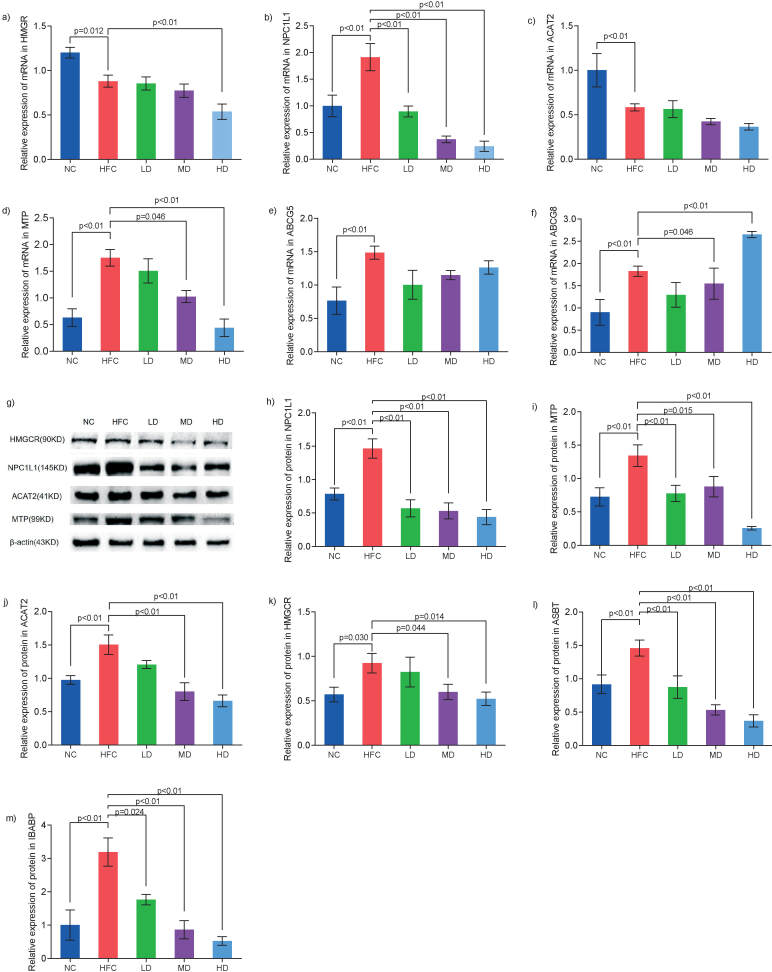
Influence of *Sparassis latifolia* polysaccharides on the expression of genes and proteins involved in cholesterol metabolism in the jejunum and ileum: a-f) the mRNA expression in HMGCR, NPC1L1, ACAT2, MTP, ABCG5 and ABCG8 in the jejunum, g) protein expression of different genes in the jejunum determined by Western blot analysis, h-k) the quantified expression of proteins in NPC1L1, MTP, ACAT2 and HMGCR, l and m) the expression of mRNA in ASBT and IBABP in the ileum. NC=normal control group, HFC=high-fat and high-cholesterol diet group, LD=group receiving a low dose of polysaccharides, MD=group receiving a medium dose of polysaccharides, HD=group receiving a high dose of polysaccharides. The significant differences were at p<0.05, and highly significant differences were at p<0.01

To investigate the molecular mechanism of *S. latifolia* polysaccharides to modulate cholesterol metabolism, mRNA and protein abundances of HMGCR, NPC1L1, ACAT2, MTP and ABCG5/8 in jejunum, and mRNA abundances of *ASBT* and *IBABP* in ileum were determined. As shown in Fig. 5, the expression of NPC1L1, MTP and ABCG5/8 were significantly up-regulated in HFC group compared with the NC group (p<0.05). Compared with the HFC group, the mRNA expressions of *HMGCR*, *NPC1L1*, *ACAT2* and *MTP* in three groups treated with *S. latifolia* polysaccharides decreased in a dose-dependent manner (Figs. 5a−5d), and the mRNA expression of *HMGCR*, *NPC1L1* and *MTP* in HD group were significantly (p<0.05) down-regulated by 39.0, 87.3 and 71.6%, respectively (Figs. 5a, 5b and 5d). However, the ABCG8 mRNA expression was significantly (p<0.05) up-regulated by 45.2% (Fig. 5f). At translational level (Fig. 5g), the protein abundances of HMGCR, NPC1L, ACAT2 and MTP were significantly (p<0.05) up-regulated in the HFC group compared with those in the NC group. Compared with HFC group, *S. latifolia* polysaccharides significantly reduced the protein expressions of NPC1L1 and MTP in all treated groups (Figs. 5h and 5i), ACAT2 in MD and HD groups (Fig. 5j), and HMGCR in HD group (p<0.05) (Fig. 5k).

As shown in Figs. 5l and 5m, expression of *ASBT* and *IBABP* mRNA levels significantly (p<0.05) increased in the HFC group compared with the NC group. Compared with the HFD group, the mRNA expressions of *ASBT* and *IBABP* in the groups treated with *S. latifolia* polysaccharide decreased in a dose-dependent manner. The *ASBT* mRNA expression in the LD, MD and HD groups were significantly (p<0.05) down-regulated by 41.0, 63.5 and 74.9%, respectively (Fig. 5l). The expression of IBABP mRNA in LD, MD, and HD groups were significantly (p<0.05) reduced by 44.7, 73.0 and 83.6%, respectively (Fig. 5m).

The *S. latifolia* polysaccharides can alleviate the intestinal cholesterol metabolism disorder induced by high-fat and high-cholesterol diet by regulating the expressions of genes and proteins related to cholesterol metabolism in the small intestine. HMGCR is the rate-limiting enzyme in cholesterol synthesis and an important target in the synthesis process. Studies have found that inducing degradation of HMGCR protein could effectively reduce cholesterol content (*28*), which was an effective method for the treatment of hypercholesterolaemia. Our data indicated that the expression of protein HMGCR highly increased in HFC group compared with the NC group, but the mRNA and the protein expressions significantly decreased in the HD group compared with the HFC group, which indicated that *S. latifolia* polysaccharides decreased the *de novo* biosynthesis of cholesterol by inhibiting the gene transcription and the protein abundance in enzymes involved in the cholesterol biosynthesis. Similar results were obtained in a previous study (*29*). The cholesterol absorption is governed by two types of transporters, namely, NPC1L1 and ABCG5/8. The former is an influx transporter responsible for channelling the cholesterol from the intestine lumen into enterocytes, whereas the latter is the efflux transporter responsible for shuttling some unesterified cholesterol back to the lumen for excretion (*30*). As a key target for regulating cholesterol absorption, NPC1L1 can work with ACAT2 and MTP to regulate the absorption and transport of cholesterol in intestinal epithelial cells, during which ACAT2 can promote intracellular sterol esterification in the enterocytes, whereas MTP is responsible for the chylomicron assembly. Studies have found that NPC1L1 or ACAT2 knockout mice significantly reduced cholesterol absorption capacity and could effectively resist hypercholesterolaemia and improve atherosclerosis (*31*). The clearance of MTP could significantly increase intestinal triglyceride and cholesterol amounts and reduce transport with chylomicrons (*32*). Our results indicated that the mRNA expressions of *NPC1L1* and *ACAT2* were significantly up-regulated in the HFC group compared with those in the NC group. However, compared with the HFC group, *S. latifolia* polysaccharides significantly decreased the expressions of *NPC1L1*, *ACAT2* and *MTP* mRNA in a dose-dependent manner and the expressions of NPC1L1, ACAT2 and MTP protein were significantly down-regulated in the HD group, which is in agreement with the previous report (*33*). In addition, our results also indicated that the mRNA of *ABCG8* in the HD group was overexpressed compared with the HFC group, which was consistent with a previous report (*34*), where they established that cholesterol excretion increased by increasing the expression of *ABCG8* genes. It is well known, under physiological conditions, that there are some transport pathways for nutrition and endogenous substances to traverse the intestinal epithelium. Among these, the intestinal bile acid pathway is a specific and highly effective delivery route involving ASBT-mediated cell entry and cytosolic IBABP-guided intracellular trafficking (*35*). In this study, the mRNA expressions of *ASBT* and *IBABP* were remarkably down-regulated in the HD group compared with the HFC group, which may be because *S. latifolia* polysaccharides can inhibit the reabsorption of bile acids and promote their excretion in faeces. Inhibition of ASBT and IBABP increased the excretion of bile acids in faeces, promoted the conversion of liver cholesterol to bile acids, and maintained the balance of enterohepatic circulation (*36*), which was consistent with the increase of bile acids in faeces. To sum up, *S. latifolia* polysaccharides play an important role in improving the intestinal cholesterol metabolism disorder caused by high-fat and high-cholesterol diet and maintaining intestinal homeostasis by influencing the key targets of intestinal cholesterol and bile acid metabolism, thereby inhibiting the synthesis, absorption and transport of cholesterol, but promoting its excretion, and also hindering the reabsorption of bile acids.

### Influence of S. latifolia polysaccharides on the intestinal microbiota of rats fed high-fat and high-cholesterol diet

High-throughput sequencing technology was used to investigate the impact of *S. latifolia* polysaccharides on the gut microbiota of rats. The results of α-diversity analysis are shown in Figs. 6a−6e. The significant decrease in the number of species was observed (Fig. 6a), while Chao1 and ACE indices decreased in the HFC group (HFC *vs* NC, p<0.05) and then remained similar. Compared with the HFC group, the number of observed species and Simpson’s diversity index were significantly reduced in HD group, while Shannon index was remarkably decreased in MD and HD groups. The principal coordinate analysis (PCoA), non-metric multidimensional scaling (NMDS), and principal component analysis (PCA) based on weighted UniFrac were further performed to estimate the difference of gut microbiota profiles among the five communities (NC, HFC, LD, MD and HD groups) (Figs. 6f−6h). The analysis of PCoA, NMDS and PCA plot showed that the samples in the NC and HFC groups and the groups treated with *S. latifolia* polysaccharides were apparently clustered separately, which indicated that *S. latifolia* polysaccharides induced significant changes in the composition of gut microbiota.

**Fig. 6a-h:**
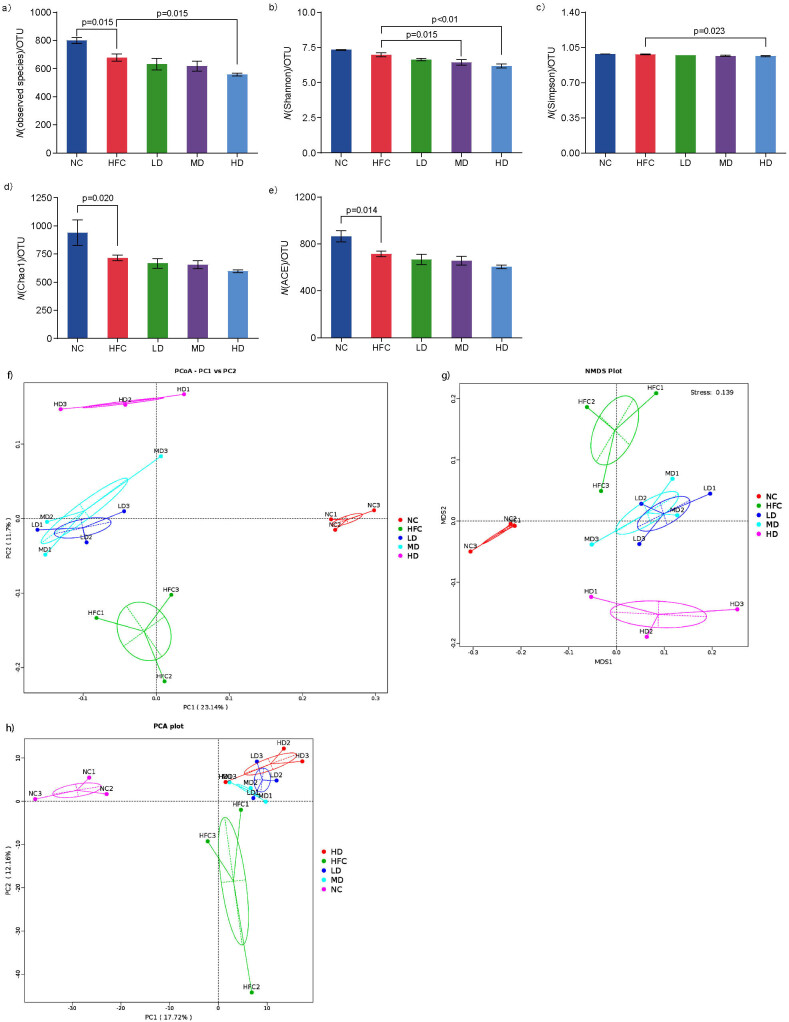
Influence of *Sparassis latifolia* polysaccharides on the intestinal microflora of rats: a-e) α- diversity of intestinal microorganisms (number of observed species and Shannon, Simpson's, Chao 1 and ACE indices respectively), f-h) different gut microbiota profiles (PCoA, NMDS and PCA). NC=normal control group, HFC=high-fat and high-cholesterol diet group, LD=group receiving a low dose of polysaccharides, MD=group receiving a medium dose of polysaccharides, HD=group receiving a high dose of polysaccharides, OTU=operational taxonomy unit, ACE=abundance-based coverage estimator. The significant differences were at p<0.05, highly significant differences were at p<0.01

The relative abundance of intestinal microbiota at the phylum level indicated that *Firmicutes*, *Bacteroidetes*, *Verrucomicrobia* and *Proteobacteria* were the most abundant in all experimental groups, and *Firmicutes* and *Bacteroidetes* accounted for approx. 75% of the total intestine microbes (Figs. 6i and 6j). Compared with the NC group, *Firmicutes* significantly (p<0.05) increased, while *Bacteroidetes* significantly decreased in the HFC group. Treatment with *S. latifolia* polysaccharides significantly (p<0.05) increased the relative abundance of *Bacteroidetes*, but reduced the content of *Firmicutes*. Moreover, the ratio of *Firmicutes* to *Bacteroidetes* in MD and HD groups significantly decreased compared with the HFC group (p<0.05).

**Fig. 6i-j:**
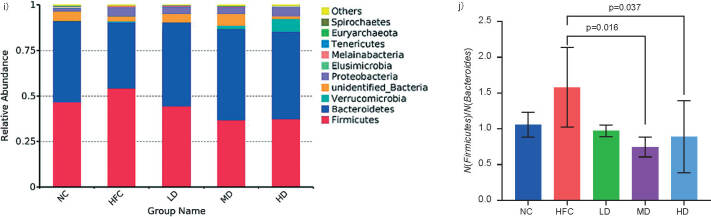
Influence of *Sparassis latifolia* polysaccharides on the intestinal microflora of rats: relative abundance of species at the phylum level, and the ratio of *Firmicutes* to *Bacteroidetes*, respectively. NC=normal control group, HFC=high-fat and high-cholesterol diet group, LD=group receiving a low dose of polysaccharides, MD=group receiving a medium dose of polysaccharides, HD=group receiving a high dose of polysaccharides. The significant differences were at p<0.05, highly significant differences were at p<0.01

In order to further explore the effects of *S. latifolia* polysaccharides on the changes of the intestinal microbiota composition in rats fed high-fat and high-cholesterol diet, the top 35 dominant microbiota in the intestine at the genus level were selected to construct a species abundance cluster heatmap. As shown in Fig. 6k, compared to the NC group, the relative abundance of beneficial bacteria including *Bacteroides*, *Blautia*, *Ruminiclostridium*, *Elusimicrobium* and *Alistipes* in the HFC group was reduced but the differences were not significant. However, the relative abundance of harmful bacteria or conditional pathogenic bacteria in the HFC group increased, such as *Desulfovibrio*, *Peptococcus*, *Moraxella*, unidentified species from *Lachnospiraceae* family, and unidentified species from *Enterobacteriaceae* family, of which the relative abundance of *Peptococcus* in the HFC group significantly increased (p<0.05). Compared with the HFC group, the relative abundance of the above bacterial genera in three groups treated with *S. latifolia* polysaccharides was revitalized, and the relative abundance of *Parabacteroides*, *Parasutteerella*, *Butyricicoccus* and *Alloprevotella* in the HD group significantly increased (p<0.05). The comparison of the microbiota with significant differences at the genus level in each group is shown in Fig. 6l-q. The HFC group exhibited distinct increases in the relative abundance of *Parasutterella* and *Peptococcus* as compared to NC group (p<0.05), and slight increases in the relative abundance of *Butyricicoccus*, *Parabacteroides* and *Alloprevotella*, but the difference was not significant. Compared with the HFC group, the relative abundance of *Bacteroides* and *Alloprevotella* significantly increased in the LD group (p<0.05). The relative abundance of *Butyricicoccus* distinctly increased in the MD group (p<0.05). The rats in HD group showed distinct increases in the relative abundance of *Bacteroides*, *Butyricicoccus*, *Parabacteroides* and *Alloprevotella* (p<0.05), but remarkable decreases in the relative abundance of *Bacteroides*, *Alloprevotella* and *Peptococcus* (p<0.05).

**Fig. 6k:**
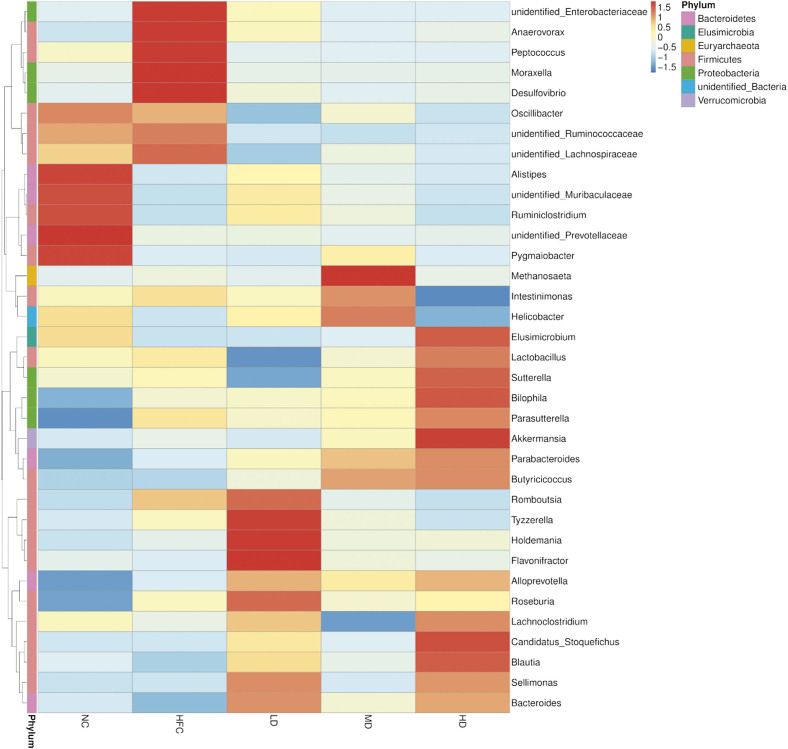
Influence of *Sparassis latifolia* polysaccharides on the intestinal microflora of rats: heatmap of species abundance clustering at genus level. NC=normal control group, HFC=high-fat and high-cholesterol diet group, LD=group receiving a low dose of polysaccharides, MD=group receiving a medium dose of polysaccharides, HD=group receiving a high dose of polysaccharides

**Fig. 6l-q:**
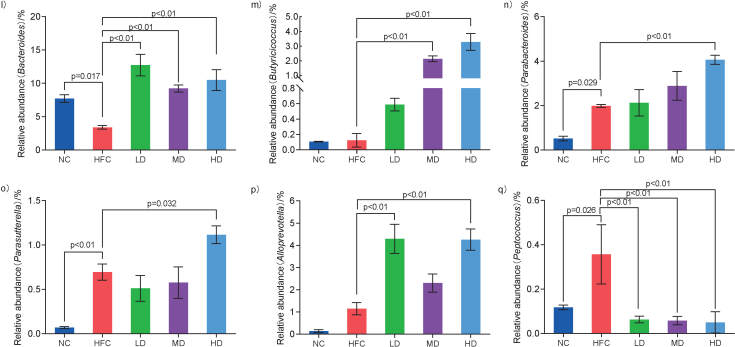
Influence of *Sparassis latifolia* polysaccharides on the intestinal microflora of rats: significant differences of intestinal microflora at genus level. NC=normal control group, HFC=high-fat and high-cholesterol diet group, LD=group receiving a low dose of polysaccharides, MD=group receiving a medium dose of polysaccharides, HD=group receiving a high dose of polysaccharides. The significant differences were at p<0.05, highly significant differences were at p<0.01

### Influence of S. latifolia polysaccharides on SCFAs in caecal content of rats fed high-fat and high-cholesterol diet

**Fig. 7:**
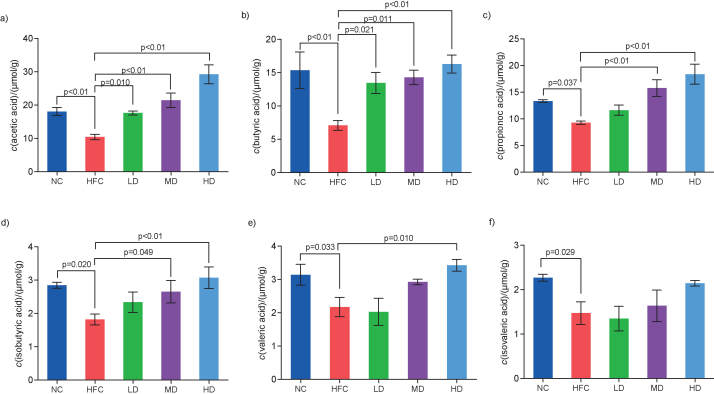
Influence of *Sparassis latifolia* polysaccharides on the concentrations of the short-chain fatty acids (SCFAs) in the rat cecal content: a) acetic, b) butyric, c) propionic, d) isobutyric, e) valeric and f) isovaleric acids. NC=normal control group, HFC=high-fat and high-cholesterol diet group, LD=group receiving a low dose of polysaccharides, MD=group receiving a medium dose of polysaccharides, HD=group receiving a high dose of polysaccharides. The significant differences were at p<0.05, highly significant differences were at p<0.0

As shown in Fig. 7, all SCFA concentrations detected in the HFC group were significantly reduced compared with the NC group (p<0.05). Compared with the HFC group, the concentrations of SCFAs in the groups treated with *S. latifolia* polysaccharides increased in a dose-dependent manner. The concentrations of acetic and butyric acids in three groups treated with *S. latifolia* polysaccharides significantly (p<0.05) increased (Figs. 7a and 7b), and the propionic, isobutyric and valeric acids in the HD group significantly (p<0.05) increased, by 98.2, 68.8 and 57.6%, respectively (Figs. 7c−7e).

### Functional prediction of gut microbiota induced by S. latifolia polysaccharides

**Fig. S1:**
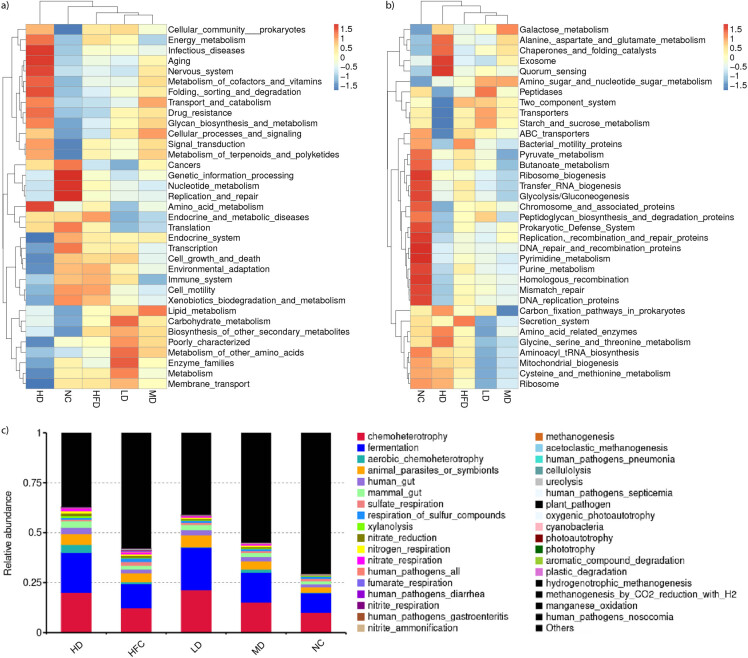
Functional prediction analysis of gut microbiota in rats treated with *Sparassis latifolia* polysaccharides. The functional analysis based on KEGG database (*24*) at: a) level 2 and b) level 3; c) relative abundance of functional categories in gut microbiota among the five groups as predicted by FAPROTAX (*25*). NC=normal control group, HFC=high-fat and high-cholesterol diet group, LD=group receiving a low dose of polysaccharides, MD=group receiving a medium dose of polysaccharides, HD=group receiving a high dose of polysaccharides

Based on the results of 16s RNA, we further used Tax4Fun (*23*) to predict the functional composition of a metagenome using KEGG databases (*24*) as reference genomes to investigate the impact of *S. latifolia* polysaccharides treatment on the metabolism pathway. The results of the analysis of the relative abundance of KEGG on level 2 demonstrated that the treatment with *S. latifolia* polysaccharides up-regulated the energy metabolism, amino acid metabolism, glycan biosynthesis and metabolism, lipid metabolism and carbohydrate metabolism, and down-regulated the nucleotide metabolism, xenobiotics biodegradation and metabolism (Fig. S1a). The analysis of the relative abundance of KEGG on level 3 showed that the treatment with *S. latifolia* polysaccharides promoted glycolysis/gluconeogenesis, ATP-binding cassette (ABC) transporters, peptidoglycan biosynthesis and protein degradation, and down-regulated alanine, aspartate and glutamate metabolism (Fig. S1b). A functional prediction analysis based on the FAPROTAX database (*25*) shows that most of the detected bacterial genera are involved in the processes of chemoheterotrophy and fermentation, which indicates that the differences in biochemical processes might explain the effects of gut microbiota on intestinal function (Fig. S1c).

### Correlation analysis of the dominant intestinal microbiota and cholesterol metabolism biomarkers

**Fig. 8:**
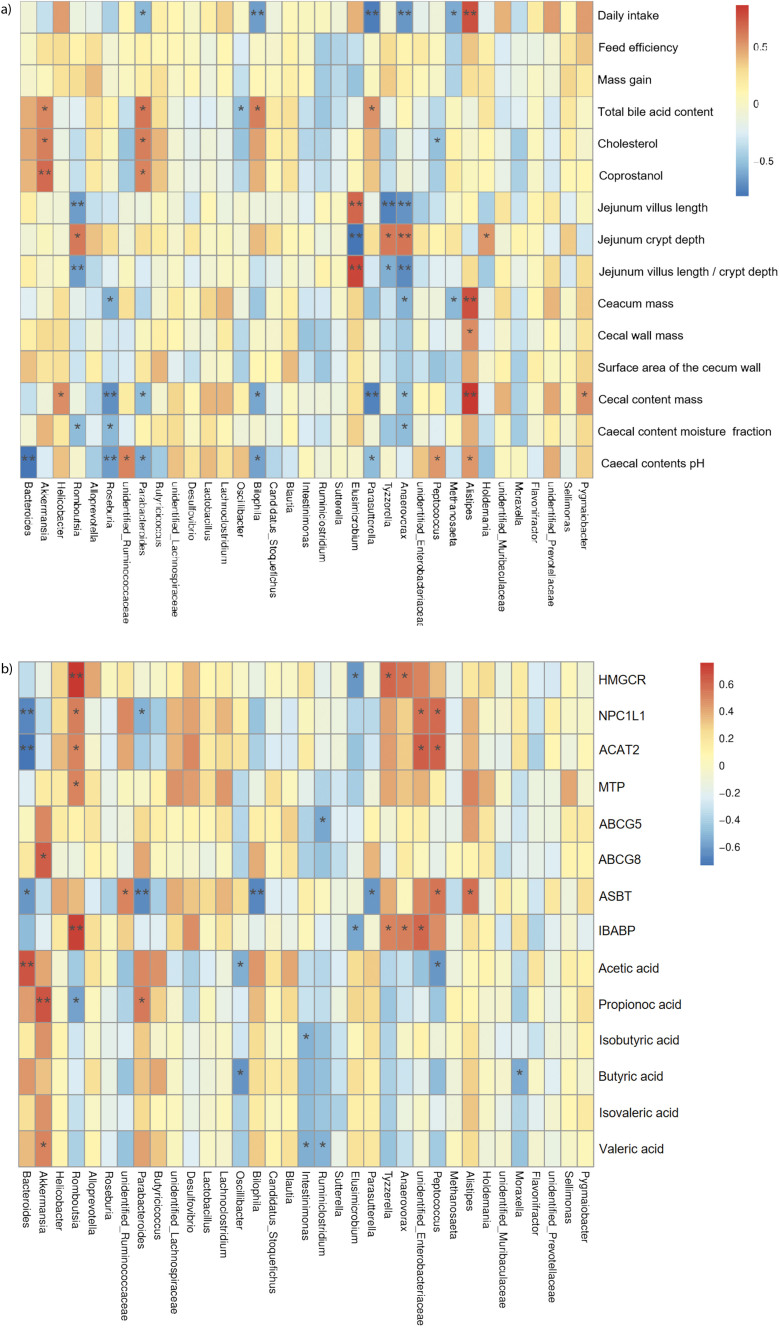
The correlation analysis of gut microbiota and markers of cholesterol metabolism: a) Spearman's correlations between key genera and cholesterol metabolism phenotype, and b) Spearman's correlations between key genera and cholesterol metabolism parameters and gut microbiota-generated metabolites Note: Legend colours represent R-value of Spearman's correlation. *Significant at p<0.05, **significant at p<0.01

The Spearman’s correlation analysis showed that some biomarkers associated with cholesterol metabolism were significantly correlated with the abundance of specific intestinal microbiota. Bacteroides had a negative correlation with the pH of caecal content (Fig. 8a), NPC1L1 and ACAT2 expression, and a positive correlation (p<0.01) with acetic acid concentration (Fig. 8b). *Akkermansia* had a positive correlation (p<0.01) with the contents of total bile acid, cholesterol and coprostanol (Fig. 8a), ABCG8 expression (p<0.05) (Fig. 8b), the concentrations of propionic (p<0.01) and valeric (p<0.05) acids (Fig. 8b). *Roseburia* had a negative correlation with the pH of caecal content (p<0.01). *Parabacteroides* had a positive correlation with the contents of total bile acid, cholesterol, coprostanol and the concentration of propionate acid, and a negative correlation (p<0.05) with the pH of caecal content (Fig. 8a). *Oscillibacter* had a negative correlation (p<0.05) with the concentrations of total bile acid (Fig. 8a), acetic and butyric acids (Fig. 8b). *Peptoccoccus* had a negative correlation with the mass fraction of cholesterol (Fig. 8a) and the concentration of acetic acid (Fig. 8b), and a positive correlation with the pH of the caecal content (Fig. 8a) and NPC1L1 and ACAT2 expression (Fig. 8b) (p<0.05).

On the one hand, the increase of bile acid concentration in faeces may be caused by the presence of viscous polysaccharides in the small intestine, which has been shown to prevent at least some bile salts from being re-absorbed into the enterohepatic circulation, resulting in excess excretion of bile salts in faeces (*37*). β-glucan of *S. latifolia* polysaccharides can be adsorbed and combined with cholesterol and bile acids to increase the viscosity of chyme, increase gastrointestinal motility, reduce the time of chyme in the intestine, and reduce the contact between the intestinal epithelial cells and cholesterol. At the same time, it also reduces the reabsorption of bile acids in the terminal ileum, so that more cholesterol is converted into bile acids, and stool cholesterol and bile acid contents increase (*38*). On the other hand, the change of bile acid excretion in faeces could be related to the gut microbiota. Our results showed that the bile acid concentration was positively correlated with the relative abundance of *Akkermansia* and *Parabacteroides*, but negatively with the relative abundance of *Oscillibacter* and *Peptoccoccus*. Moreover, the relative abundance of *Akkermansia* and *Parabacteroides* in the groups treated with *S. latifolia* polysaccharides increased, while the relative abundance of *Oscillibacter* and *Peptoccoccus* decreased, which was consistent with the changes in bile acid concentration. The increase of bile acid excretion in faeces could reduce cholesterol mass fraction by disrupting the formation of micelles, leading to a reduced ability to solubilize cholesterol (as well as monoglycerides and fatty acids) and consequently reducing cholesterol absorption in the body. In addition, the surface area of the caecal wall in the groups treated with *S. latifolia* polysaccharides significantly increased, while the pH of the caecal content significantly decreased (the groups treated with *S. latifolia* polysaccharides *vs* HFC, p<0.05). The increase in the volume of the caecal content and the surface area of the caecal wall may be caused by the *S. latifolia* polysaccharides which are not digested and absorbed by the small intestine so they enter the caecum to absorb water and swell (*39*). The lower pH in the caecum may be related to the increase of SCFA concentration produced during the fermentation of *S. latifolia* polysaccharides by the intestinal microbiota (*40*), confirmed by the positive correlation between the pH in the caecum and the relative abundance of specific intestinal microbiota, such as *Bacteroides*, *Roseburia* (p<0.01) and *Parabacteroides* (p<0.05), of which *Bacteroides* had a positive correlation with the acetic acid concentration (p<0.01) and *Parabacteroides* with the propionic acid concentration (p<0.01). The improvement of the intestine morphology and environment in rats fed high-fat and high-cholesterol diet after treatment with *S. latifolia* polysaccharides is due to the functional characteristics of β-glucan, the main component of the polysaccharides, such as water-holding swelling, strong binding ability, and the sensitivity of bacterial degradation and fermentation.

Generally, the homeostasis of cholesterol is regulated by its intestinal absorption, intracellular synthesis and bile excretion. Long-term intake of high-fat and high-cholesterol diets can cause damage to the structure of the intestinal tract, disorder of cholesterol metabolism and imbalance of the intestinal microecology. An upset in cholesterol homeostasis can increase the risk factors of heart diseases and type II diabetes (*40*). Recently, polysaccharides from edible fungi have gained much attention because of their potential health-promoting effects. In this study, polysaccharides were extracted from *S. latifolia* to study their mechanism of regulation of the intestinal cholesterol metabolism of rats fed high-fat and high-cholesterol diet.

Intestinal microbiota can affect the growth of the body and directly participate in various metabolic processes, including immune regulation, neuromodulation and the metabolism of fat, amino acids and carbohydrates in the body, and the imbalance of the intestinal microflora can lead to various diseases (*41*). A number of studies have demonstrated the correlative relationship of lipid metabolism and gut microbiota composition (*41*). In our study, the number of *Firmicutes* significantly increased, while the number of *Bacteroidetes* remarkably decreased in HFC group compared with the NC group, which could be explained by the fact that high-fat diet had a greater effect on the bacterial structure at the phylum level, increased the abundance of *Firmicutes* and decreased that of *Bacteroidetes* (*42*). Similar result was obtained in a previous study which found that the relative abundance of *Firmicutes* in the intestinal flora of obese people was relatively high, while the relative abundance of *Bacteroidetes* was relatively low. In addition, the increased ratio of *N*(*Firmicutes*)/*N*(*Bacteroidetes*) would cause the intestinal flora to obtain energy more effectively, promote the synthesis of fat and cholesterol, and result in diseases such as hyperlipidaemia and lipid metabolism disorders (*42*). The high fat and cholesterol diet in this study could significantly increase the *N*(*Firmicutes*)/*N*(*Bacteroidetes*) ratio by 52.2%, which is consistent with previous findings (*43*). Consumption of polysaccharides can reverse the ratio of *Firmicutes* and *Bacteroides* (*43*). Treatment with *S. latifolia* polysaccharides significantly reduced the content of *Firmicutes* and resulted in a significant reduction of the *N*(*Firmicutes*)/*N*(*Bacteroidetes*) ratio in the MD and HD groups, which indicated that the polysaccharides had the potential to reduce the absorption of excess energy by the host from the diet by adjusting the relative abundances of *Firmicutes* and *Bacteroidetes* and regulating the body's metabolism. The decrease of the *N*(*Firmicutes*)/*N*(*Bacteroidetes*) ratio might result from β-(1→3)-d-glucan, the major structural unit of the polysaccharides, which has been believed to stimulate and increase the content of probiotics in the intestine (*40*).

In addition to the shift at the phylum level, *S. latifolia* polysaccharides altered some important communities in genus level related to cholesterol and bile acid metabolism. The analysis of the changes in the genus level found that *S. latifolia* polysaccharides could significantly increase the relative abundance of intestinal microbiota related to cholesterol and bile acid metabolism including *Bacteroides*, *Butyricicoccus*, *Parabacteroides*, *Parasutterella*, *Alloprevotella*. *Bacteroides* have a single exemplary polysaccharide utilization site, which can degrade *S. latifolia* polysaccharides and accelerate the conversion of cholesterol to bile acid, thereby promoting the metabolism (*42*). *Butyricicoccus* can ferment *S. latifolia* polysaccharides to produce butyric acid, consistent with the increase of butyric acid concentration in MD and HD groups, which provides energy for the body and promotes the development of intestinal epithelial cells. *Parabacteroides* has the function of converting bile acid and producing SCFAs, and the increase of its relative abundance can reduce obesity and enhance intestinal integrity (*44*), which was proved by the increased SCFAs concentration and the improved mucosa structure and intestinal epithelial cells after treatment with the polysaccharides. *Parasutterella* is considered to be the core component of the intestinal microbiota, which can ferment the polysaccharides to maintain bile acid balance and participate in cholesterol metabolism (*45*). *Alloprevotella* is the dominant microbiota in the healthy intestine, and the increase of its relative abundance is negatively correlated with serum cholesterol and low-density lipoprotein cholesterol amounts (*46*). However, *S. latifolia* polysaccharides significantly inhibited the relative abundance of *Peptococcus* and *Oscillibacter*. *Peptococcus*, closely associated with infectious diseases or inflammation, is considered to be a conditional pathogen (*47*). *Oscillibacter* is known as the fat bacteria in the intestine, and its relative abundance is negatively correlated with indicators related to cholesterol metabolism, and the result is the same as in the previous report (*47*).

The functional metabolites of the intestinal microbiota also play an important role in the health of the body. At the same time, the SCFAs produced by the fermentation of polysaccharides in the gut cannot only lower the pH of the intestines and inhibit harmful microbiota, but also provide energy for the body, regulate a variety of transcription factors and affect energy metabolism (*48*). The high-fat and high-cholesterol diets reduced the concentration of SCFAs in faeces, while *S. latifolia* polysaccharides restored the concentration of SCFAs in a dose-dependent manner. This may be because the polysaccharides are fermented by anaerobic bacteria in the caecum to produce a large number of SCFAs such as acetic, propionic and butyric acids. Acetic acid can prevent diet-induced obesity and insulin resistance (*49*). The increased acetic acid concentration after the treatment with *S. latifolia* polysaccharides was positively correlated with the relative abundance of *Bacteroidetes* (p<0.01), confirmed by the fact that acetate is predominantly produced by the *Bacteroidetes* (*50*). Propionic acid can promote the secretion of peptide YY and glucagon-like peptide-1, and reduce energy intake. The increase of propionic acid concentration in HD group was positively correlated with the relative abundance of *Akkermansia*, whose main metabolite is propionic acid, which can improve the inflammatory response of obese and diabetic patients (*51*). Butyric acid can prevent the absorption of intestinal cholesterol in apolipoprotein E-deficient mice and reduce atherosclerosis (*52*). Moreover, butyric acid is the main energy source used by the colon cells and is widely produced by *Clostridium* in the *Firmicutes* phylum, consistent with the increased relative abundance of *Firmicutes* in the groups treated with *S. latifolia* polysaccharides. Therefore, the increase of specific SCFA concentration in this study may have an important role in maintaining the intestinal microbes. In addition, negative correlation was observed between the expression of HMGCR and the concentrations of propionic and butyric acids, indicating that the inhibition of cholesterol synthesis after the treatment with *S. latifolia* polysaccharides might be mediated by gut microbiota metabolites.

The intake of *S. latifolia* polysaccharides affects predictive function and metabolism of intestinal flora. The results showed that *S. latifolia* polysaccharides could improve the energy metabolism, lipid metabolism, carbohydrate metabolism and amino acid metabolism. By changing the intestinal microbial composition, more *S. latifolia* polysaccharide products are formed by the intestinal microbial fermentation, which thereby improves cholesterol metabolism disorder caused by high fat and high cholesterol diet, consistent with the production of short-chain fatty acids. ABC transport system is one of the main mechanisms by which bacteria degrade polysaccharides. It is a common polysaccharide degradation system in *Firmicutes* and *Bifidobacterium.* The changes of *Firmicutes* and *Bifidobacterium* were consistent with that of ABC transport. *Bacteroides* have several carbohydrate metabolic pathways and encode diverse degradative enzymes including glycoside hydrolases, polysaccharide lyases and carbohydrate esterases, which confer strong ability to metabolize carbohydrates. The improvement of carbohydrate metabolism was proved by the increase of relative abundance in *Bacteroides*. Soluble dietary polysaccharides inhibit mass gain, fat accumulation and promote the catabolism of cholesterol by increasing energy consumption and regulating the intestinal microbiota (*53*), which is consistent with the changes in energy metabolism and lipid metabolism.

## CONCLUSIONS

In conclusion, our study showed the regulatory effect of *Sparassis latifolia* polysaccharides on the intestinal cholesterol metabolism in rats fed high-fat and high-cholesterol diet by improving the morphological structure and physiological indices of the intestinal tract, reversing the expressions genes involved in cholesterol and bile acid metabolism, and increasing the relative abundance of the dominant intestinal microbiota and the concentration of the short-chain fatty acids. *S. latifolia* polysaccharides were involved in lipid and carbohydrate metabolism, ameliorated the metabolic production and metabolic pathways related to the changes caused by high-fat and high-cholesterol diet, and had a beneficial effect on the cholesterol metabolism. This will provide a theoretical basis for developing *S. latifolia* polysaccharides as a functional food material for regulating cholesterol metabolism. However, the relationship between the beneficial microbiota and their functional metabolites or the metabolism of intestinal cholesterol and bile acids concentration needs further investigation.

## Appendix

**Table S1:** Composition of basic diet for rats

Ingredient	*w*/%
Flour	15.2
Corn	45
Soybean meal	20
Bran	9
Fish meal	3
Yeast	2
Stone powder	1.5
Dicalcium phosphate	1.3
Salt	0.5
Cooking oil	2
Trace elements	*w*/(g/100 kg) 20
Vitamins	10
Cod liver oil	50

**Table S2:** Primer pairs used for quantitative real-time polymerase chain reaction (qRT-PCR)

Target gene	Primer sequence (5’-3’)	Size/bp	GenBank (*15*) number
β-actin	F: TGCTATGTTGCCCTAGACTTCG	240	NM_031144
	R: GTTGGCATAGAGGTCTTTACGG		
HMGCR	F: TGCGTGTCCCTGGTCCTA	120	NM_013134.2
	R: TTGGGTTACTGGGTTTGG		
NPC1L1	F: CTCACGGCCAACCAGACTCT	291	NM_001002025.1
	R: TTCCTTCAAGAAAGCCTCCTCC		
ACAT2	F: ACCCCGTCGTCATCATCTCAG	150	NM_001006995.1
	R: TGACCTCGGACACCTCTTCTG		
MTP	F: GTCACGATAACGGCTGTCAATG	301	NM_001107727.1
	R: GCTGAGCCTGGTAGGTGACTTT		
ABCG5	F: GCAGGGACCAGTTCCAAGAC	156	NM_053754.2
	R: TCAGGACTGCCTCTACCTTCTTG		
ABCG8	F: TGGCTACCCTTGTCCTCGCTA	129	NM_130414.2
	R: AGGAACAAGGCTGCAAGTAATCG		
ASBT	F: GTTGCCTTGGAAACTGGAATGC	259	NM_017222.2
	R: GAAATCCCTTGTTTGTCTCCTGG		
IBABP	F: CAAGTTCACCATTGGCAAAGAA	183	NM_017098.1
	R: ACACGCTCGTAGGTCACATCC		
